# Unconventional treatment of inoperable biliary IPMN with an oesophageal stent in the common bile duct: case report

**DOI:** 10.1177/26317745231183311

**Published:** 2023-07-31

**Authors:** Antti Siiki, Anne Antila, Yrjö Vaalavuo, Johanna Ronkainen, Irina Rinta-Kiikka, Johanna Laukkarinen

**Affiliations:** Department of Gastroenterology and Alimentary Tract Surgery, Tampere University Hospital, Teiskontie 35, PO BOX 2000, 33521 Tampere, Finland; Department of Gastroenterology and Alimentary Tract Surgery, Tampere University Hospital, Tampere, Finland; Department of Gastroenterology and Alimentary Tract Surgery, Tampere University Hospital, Tampere, Finland; Department of Radiology, Imaging Centre and Pharmacy, Tampere University Hospital, Tampere, Finland; Department of Radiology, Imaging Centre and Pharmacy, Tampere University Hospital, Tampere, Finland; Department of Gastroenterology and Alimentary Tract Surgery, Tampere University Hospital, Tampere, Finland; Faculty of Medicine and Health Technology, Tampere University, Tampere, Finland

**Keywords:** biliary intraductal neoplasms, case report, cholangiocarcinoma, cholangiopancreatography, endoscopic retrograde

## Abstract

Biliary intraductal papillary mucinous neoplasm (IPMN) is a rare biliary neoplasia preferably treated with oncologic resection. Endoscopic radio frequency (RF) ablation may be used as a palliative measure. We present a rare case, where heavy co-morbidities prevented surgery. Continuous mucus production caused recurrent episodes of severe cholangitis. Several ERCPs (endoscopic retrograde cholangio pancretography) were necessary due to recurrent biliary obstruction. RF ablation was not effective in the dilated common bile duct without a stricture. Standard biliary stents failed due to either migration or occlusion. When other options failed, an exceptional decision was made: a covered large diameter oesophageal stent was inserted in ERCP into the bile duct to secure bile flow and stop mucus production. Digital cholangioscopy was crucial adjunct to standard ERCP in endoscopic management. The palliative treatment method was successful: there were no stent-related adverse events or readmissions for cholangitis. The follow-up in the palliative care lasted until patient’s last 10 months of lifetime.

## Introduction

The biliary tract intraductal papillary mucinous neoplasm (BT-IPMN) is a rare premalignant tumour of the biliary duct. It resembles its more common counterpart in the main pancreatic duct. It is considered an indication for surgical resection.^1^–^3^ The diagnosis is based on magnetic resonance imaging (MRI) and computed tomography (CT) imaging^4^ combined to endoscopic retrograde cholangio pancretography (ERCP) and cholangioscopy with biopsies.^5^ Endoscopic radio frequency (RF) ablation may be indicated in inoperable situations^6^ and for recurrent episodes of cholangitis due to mucus production.^7^ We present a case of a 64-year-old female palliatively treated with an extremely rarely used method^8,9^ for extra-hepatic biliary IPMN, accompanying recurrent cholangitis. Previously, there are only two case reports: one using oesophageal stent in common bile duct for biliary decompression in pancreatic cancer^8^ and another using it to control bleeding and facilitate direct cholangioscopy for stone removal.^9^

## Case report

**Figure 1.**
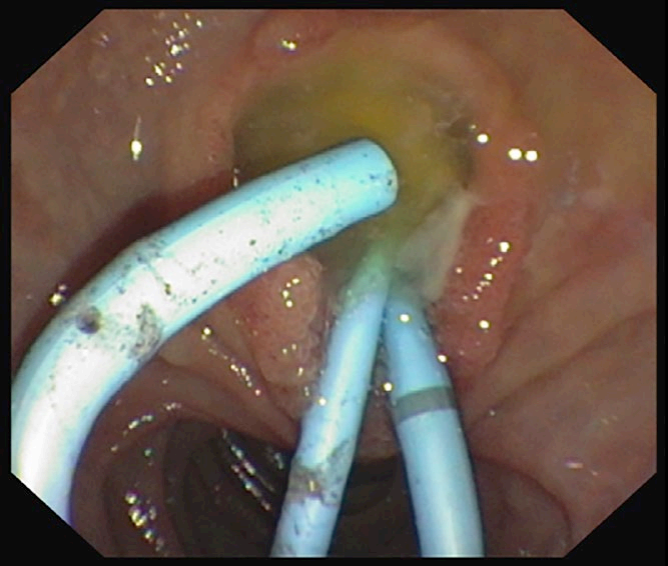
Endoscopic view of papilla where extensive amount of mucus is plugging common bile duct, occluding plastic 10F pigtail stents.

The patient was first evaluated for having elevated liver function tests. A mucus-producing BT-IPMN with low-grade dysplasia was diagnosed in ERCP and SpyGlass DS peroral-cholangioscopy (Boston Scientific, MA, USA) in late 2018. In cholangioscopy, the mucus-producing region was located in common bile duct, clearly below liver hilum. She was evaluated unfit for pancreato-duodenectomy in the multi-disciplinary team due to co-morbidities (Child-Pugh grade C cirrhosis and diabetic nephropathy). Endoscopic RF ablation was tried in early 2019, but it was found to be unsuitable due to absence of stricture in common bile duct (CBD) (Habib RF probe, Boston Sci and ERBE generator, Tuebingen, Germany). Consequently, a fully covered self-expanding metal stent (FCSEMS) was placed. A few months later, the obstructive icterus recurred. ERCP revealed wide common hepatic duct, without biliary stricture explaining also the spontaneous stent migration. After repeated ERCPs with removal of substantial amount of mucus debris and repeated insertion of plastic 10F pigtail stents in each procedure, the severe septic cholangitis persisted (Figure 1). With deteriorating liver function tests, after altogether eight ERCPs and several admissions for cholangitis, a plan for the one last time ERCP and RF was made.

**Figure 2.**
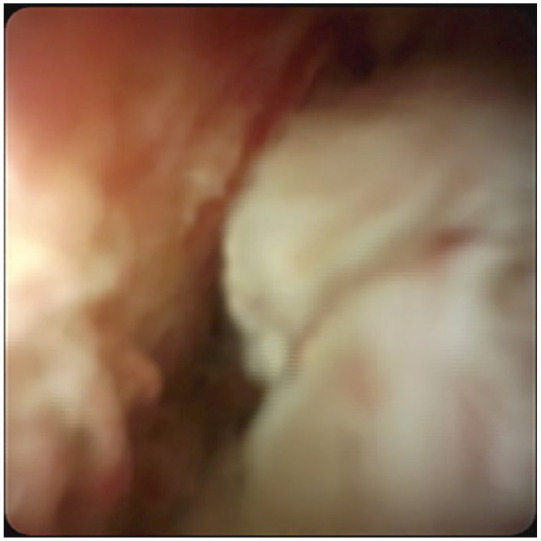
Images from SpyGlass DS cholangisopy (Boston Scientific, MA, USA) showing biliary IPMN tumour (intraductal papillary mucinous neoplasm) in the common bile duct with mucus-producing villous protrusions macroscopically resembling its more common counterpart in the main pancreatic duct (pancreatic main duct IPMN).

**Figure 3.**
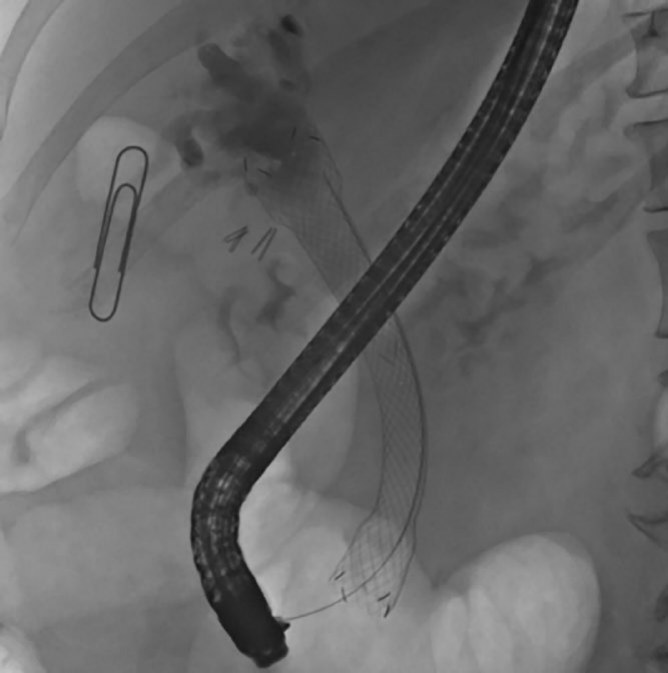
A fluoroscopy image after ERCP, SpyGlass DS cholangioscopy and eventual placement of fully covered oesophageal stent into the common bile duct to cover entire mucus-producing tumour (80 mm × 18–24 mm, Niti-S, Taewoong, Medical, Gyeonggi-Do, South-Korea).

ERCP with endoscopic RF in SpyGlass DS control was performed in September 2020 (Figure 2). Similarly to earlier attempt, the RF instrument made no proper circular contact due to wide diameter of the CBD of up to 15 mm. Only partial ablation effect to mucus-producing tumour was observed with SpyGlass DS. Obviously, it was anticipated that a standard 10 mm diameter FCSEMS would again have migrated and resulted in recurrence of extensive mucus plug, deteriorating of cholangitis and eventual liver failure with no prognostic improvement. As a palliative procedure, a fully covered Taewoong Niti-S Esophageal stent (Taewoong, Medical, Gyeonggi-Do, South-Korea) of 80 mm length and 18–24 mm diameter was introduced into the CBD through the working channel of the standard Olympus duodenoscope (Olympus Europe, Hamburg, Germany) (Figure 3). The proper positioning of the stent was controlled with SpyGlass DS to exclude stent-related obstruction in liver hilum. Mucus-producing tumour region in the CBD was now entirely sealed with the silicon covered large diameter self-expanding stent. In the following weeks, patient’s bilirubin fell from 296 to less than 50 μmol/l, to her normal preceding values. After the procedure, the continuous cycle of episodes of cholangitis ended. There were no readmissions, no stent-related pain or adverse events immediately or during the 10 months of follow-up. In this case, the stent was intended as a permanent palliative treatment with no further interventions planned. We acquired an informed consent for the treatment and anonymous case report publication from the patient. All patient data are deidentified. The ethical board review was not necessary for clinical treatment of a single case outside any research project. The case is reported according to CASE guideline.

## Discussion

Rare biliary IPMN is an indication for Whipple operation or liver resection. It bears similarities to more common counterpart, pancreatic main duct IPMN.^10^ In palliative cases, endoscopic RF ablation may be used in the biliary duct.^1,2^ SpyGlass DS cholangioscopy offers an accurate endoscopic method for diagnostic visual impression, staging of tumour extent and gaining accurate biopsies.^5,11^ It is also helpful in evaluating the instant effect after endoscopic RF ablation. By standard imaging modalities alone, such as MRI or abdominal CT, BT-IPMN may not be accurately diagnosed and staged because the differentiation, for example, from biliary sludge is not possible, as was the case here.^4^

In our patient with heavy co-morbidities, there was no possibility for surgery. Due to absence of biliary stricture, RF ablation or standard stent therapy was ineffective in widely dilated duct. As unconventional last-ditch attempt in this extremely unusual case,^8^ a large bore fully covered self-expanding oesophageal stent was placed to cover and seal the mucus-producing region. When balancing between dismal prognosis of recurrent severe cholangitis for mucus biliary obstruction and the risks of unprecedented treatment method, even inserting a stent made for oesophageal use in common bile duct by ERCP may be a viable option for the best palliative care.

## Declarations

**Ethics approval and consent to participate:** Not applicable.

**Consent for publication:** The patient provided an informed consent for the anonymous publication of their medical data and images.

**Availability of data and materials:** Not applicable.
